# *TMEM106B* analysis in diverse cohorts suggests no association with Parkinson’s disease risk

**DOI:** 10.64898/2026.09.28.26363833

**Published:** 2026-10-02

**Authors:** Henry Mauricio Chaparro-Solano, Sajanth Kanagasingam, Ziv Gan-Or, Konstantin Senkevich, the Global Parkinson’s Genetic Program (GP2)

## Abstract

Recent evidence suggests that TMEM106B expression is reduced in the brains of patients with Parkinson’s disease (PD). However, an association between rare genetic variants in TMEM106B and PD risk has not been previously established. In this work, we investigated the association between rare TMEM106B variants and PD risk. We leveraged whole-genome sequencing data from 22,010 cases and 40,550 controls across diverse ancestries from the Global Parkinson’s Genetics Program (GP2), the Accelerating Medicines Partnership in Parkinson’s Disease (AMP-PD), and the UK Biobank (UKBB). A total of 4,581 unique rare variants were identified across all groups. We found no evidence that rare coding variants in TMEM106B were associated with PD risk, either individually or through cumulative burden. These findings suggest that rare variation in TMEM106B does not play a major role in PD susceptibility across the populations studied.

## Full Text Availability

The license terms selected by the author(s) for this preprint version do not permit archiving in PMC. The full text is available from the preprint server.

